# Sphingosine-1-phosphate-Mediated Mobilization of Hematopoietic Stem/Progenitor Cells during Intravascular Hemolysis Requires Attenuation of SDF-1-CXCR4 Retention Signaling in Bone Marrow

**DOI:** 10.1155/2013/814549

**Published:** 2013-12-29

**Authors:** Kasia Mierzejewska, Yuri M. Klyachkin, Janina Ratajczak, Ahmed Abdel-Latif, Magda Kucia, Mariusz Z. Ratajczak

**Affiliations:** ^1^Stem Cell Institute, James Graham Brown Cancer Center, University of Louisville, 500 S. Floyd Street, Room 107, Louisville, KY 40202, USA; ^2^Department of Physiology, Pomeranian Medical University, Szczecin, Poland; ^3^Division of Cardiovascular Medicine, Gill Heart Institute, University of Kentucky, Lexington, KY 40536, USA

## Abstract

Sphingosine-1-phosphate (S1P) is a crucial chemotactic factor in peripheral blood (PB) involved in the mobilization process and egress of hematopoietic stem/progenitor cells (HSPCs) from bone marrow (BM). Since S1P is present at high levels in erythrocytes, one might assume that, by increasing the plasma S1P level, the hemolysis of red blood cells would induce mobilization of HSPCs. To test this assumption, we induced hemolysis in mice by employing phenylhydrazine (PHZ). We observed that doubling the S1P level in PB from damaged erythrocytes induced only a marginally increased level of mobilization. However, if mice were exposed to PHZ together with the CXCR4 blocking agent, AMD3100, a robust synergistic increase in the number of mobilized HSPCs occurred. We conclude that hemolysis, even if it significantly elevates the S1P level in PB, also requires attenuation of the CXCR4-SDF-1 axis-mediated retention in BM niches for HSPC mobilization to occur. Our data also further confirm that S1P is a major chemottractant present in plasma and chemoattracts HSPCs into PB under steady-state conditions. However, to egress from BM, HSPCs first have to be released from BM niches by blocking the SDF-1-CXCR4 retention signal.

## 1. Introduction

Hemolytic syndromes, such as sickle cell anemia (SSA) and paroxysmal nocturnal hemoglobinuria (PNH), are characterized by an increase in the number of hematopoietic stem/progenitor cells (HSPCs) circulating in peripheral blood (PB) [1–3]. However, the molecular mechanisms responsible for the process of HSPC mobilization and their egress from bone marrow (BM) into PB still are not completely understood.

In our previous work, we have demonstrated that sphingosine-1-phosphate (S1P) released in PB from lysed erythrocytes and activated platelets is a strong chemottractant for bone marrow- (BM-) residing HSPCs [4]. Based on this observation, we hypothesized that S1P released from lysed erythrocytes is a major factor responsible for egress of HSPCs from BM into PB in hemolytic syndromes. We also postulated that in PB, even under steady-state conditions, S1P creates a potent, permanent, chemotactic gradient for HSPCs, [4] which are actively retained in BM due to retention signaling involving mainly the interactions between CXCR4 receptor and stromal derived factor-1 (SDF-1) and between very late antigen-4 (VLA-4, also known as *α*_4_ *β*_1_ integrin) receptor and vascular adhesion molecule-1 (VCAM-1, also known as CD106) [5, 6].

To test the importance of changes in the S1P level in PB in the egress of HSPCs from BM, normal mice were injected with phenylhydrazine (PHZ), a compound known to induce hemolysis [7], and we evaluated the number of circulating Sca-1^+^Kit^+^Lin^−^ (SKL) HSPCs and clonogenic CFU-GM progenitors in PB. In parallel, we measured the PB level of S1P by mass spectrophotometry and the level of stromal derived factor-1 (SDF-1) by ELISA. In addition, we measured the level of free hemoglobin (Hb) as well as activation of the complement cascade (CC) by employing ELISA to detect the C5b-C9 (membrane attack complex, MAC). In addition to PHZ administration alone, in some of the experiments, we combined PHZ treatment with injection of the CXCR4 antagonist AMD3100.

We report here that hemolysis, even if it significantly elevates the S1P level in PB, requires attenuation of the CXCR4–SDF-1 axis-mediated retention of HSPCs in BM niches in order to affect mobilization of HSPCs.

## 2. Material and Methods

### 2.1. Animals

C57BL/6 mice were purchased from the National Cancer Institute (Frederick, MD USA; http://www.cancer.gov/). All mice were allowed to adapt for at least 2 weeks and used for experiments at age of 6 to 8 weeks. Animal studies were approved by the Animal Care and Use Committee of the University of Louisville (Louisville, KY, USA).

### 2.2. Treatment of Mice with PHZ and/or AMD3100

C57Bl/6 mice were injected intraperitoneally once with 40 mg/kg of PHZ [7] and, in some experiments, injected subcutaneously with 2.5 mg/kg of AMD3100.

### 2.3. Peripheral Blood Parameter Counts

Mice were bled from the retroorbital plexus to obtain leukocyte counts using Unopette Microcollection (Becton Dickinson, Rutherford, NJ, USA), and samples were run within 2 hours of collection on a Hemavet 950 analyzer as described [4].

### 2.4. FACS Analysis of SKL Cells

Six hours after PHZ injection, alone or together with AMD3100, and 1 hour after AMD3100 injection alone, PB was obtained from the vena cava (with a 25-gauge needle and 1 mL syringe containing 250 U heparin). The following monoclonal antibodies (mAbs) were employed to stain Sca-1^+^/c-Kit^+^/Lin^−^ (SKL cells): biotin-conjugated rat anti-mouse Ly-6A/E (Sca-1, clone E13-161.7), streptavidin-phycoerythrin- (PE-) Cy5-conjugated anti-mouse c-Kit (clone 2B8), and lineage markers anti-mouse CD45R/B220-PE (clone RA3-6B2), anti-mouse TCRab-PE (clone H57-597), anti-mouse TCR*γζ*–PE (clone GL3), anti-mouse CD11b-PE (clone M1/70), anti-mouse Ter119-PE (clone TER-119), and anti-mouse Gr-1-PE (clone RB6-8C5) as described [4, 8]. All mAbs were added at saturating concentrations, and the cells were then incubated for 30 minutes on ice, washed twice, resuspended in RPMI 1640 + 2% FBS, and analyzed with an LSR II flow cytometer (BD, USA).

### 2.5. Enumeration of the Number of Colony-Forming Unit-Granulocyte/Macrophage (CFU-GM) Mobilized into PB

Cells (1 × 10^6^) from PB were resuspended in 10% culture medium with 90% human methylcellulose base media supplemented with 25 ng/mL recombinant murine (rm) GM-CSF and 10 ng/mL recombinant murine (rm) IL-3. After 1 week of culture, the numbers of CFU-GM colonies were scored using an inverted microscope (Olympus, USA) [4, 8].

### 2.6. Plasma Concentration of S1P

Analysis of S1P in peripheral blood plasma was carried out using a Shimadzu UFLC coupled with an AB Sciex 4000-Qtrap hybrid linear ion trap triple quadrupole mass spectrometer in multiple reaction monitoring (MRM) mode. Detailed LC/MS/MS conditions for analysis of S1P were previously described [4].

### 2.7. Plasma Concentration of SDF-1

Plasma SDF-1 levels were evaluated by employing a sandwich enzyme-linked immunosorbent assay (ELISA) using a commercially available ELISA system (R & D Systems, Minneapolis, MN, USA) as described [4, 8].

### 2.8. Plasma Concentration of C5b-C9 (MAC Complex)

The concentration of C5b-C9 was measured by employing the commercially available, highly sensitive ELISA kit K-ASSAY (Kamiya Biomedical Company, USA), according to the manufacturer's protocol [9].

### 2.9. Statistical Analysis

Arithmetic means and standard deviations were calculated using Instat 1.14 (Graphpad, San Diego, CA, USA) software. Statistical significance was defined as *P* < 0.01. Data were analyzed using Student's *t*-test for unpaired samples.

## 3. Results

### 3.1. S1P Plasma Level Increases following PHZ Administration

**Figure 1:**
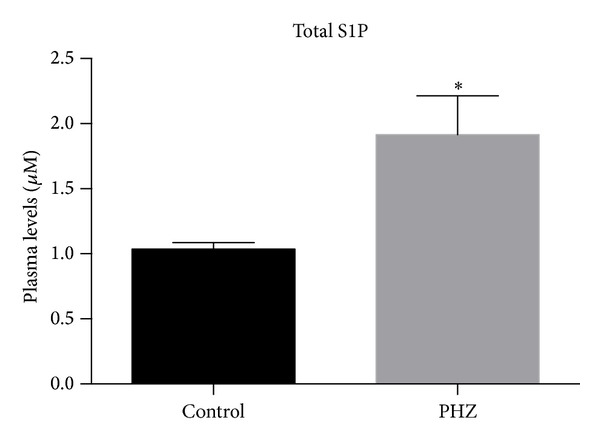
PHZ induces an increase in the total level of S1P in PB. S1P was measured by employing mass spectrophotometry in PB samples harvested at the peak of the mobilization process from mice exposed to phenylhydrazine (PHZ) and from nonmobilized control animals. The data are combined from two independent experiments with 5 animals each. *P < 0.001.

As reported previously, S1P is a potent chemoattractant for BM-residing HSPCs [4]. By employing sensitive mass spectrophotometry measurements, we observed that its level increases twofold, from ~1 *μ*M to 2 *μ*M, by 6 hours after PHZ administration (Figure 1).

### 3.2. HSPCs Are Mobilized at Negligible Levels in Response to PHZ-Induced Hemolysis

**Figure 2:**
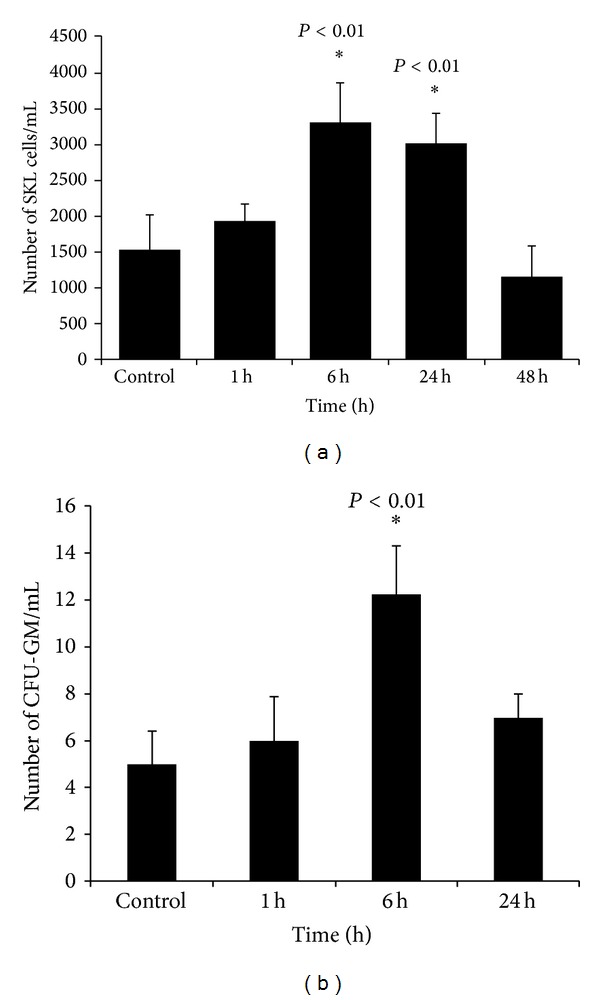
Kinetic of effect of PHZ-induced hemolysis on the mobilization of SKL cells and CFU-GM clonogenic progenitors. C57Bl/6 mice (10 mice per group) were sacrificed 1, 6, and 24 h after injection of PHZ (40 mg/kg i.p.). Control animals were injected with saline (0.9%). (a) shows the number of Sca-1^+^Kit^+^Lin^−^ (SKL) HSPCs circulating in PB (*P < 0.01) and (b) shows the number of clonogenic CFU-GM progenitors circulating in PB (*P < 0.01).

We observed that, despite a twofold increase in S1P level in PB after PHZ-induced hemolysis (Figure 1), the increase in S1P was not sufficient to mobilize significant numbers of HSPCs (Figure 2). Kinetic studies revealed that the number of circulating SKL cells and CFU-GM progenitors increased only ~2 times (Figure 2(a)) and ~2.5 times (Figure 2(b)), respectively, after PHZ-induced hemolysis, with a peak observed 6 hours after PHZ administration.

### 3.3. Synergistic Effect of PHZ + AMD3100 Mobilization of HSPCs

**Figure 3:**
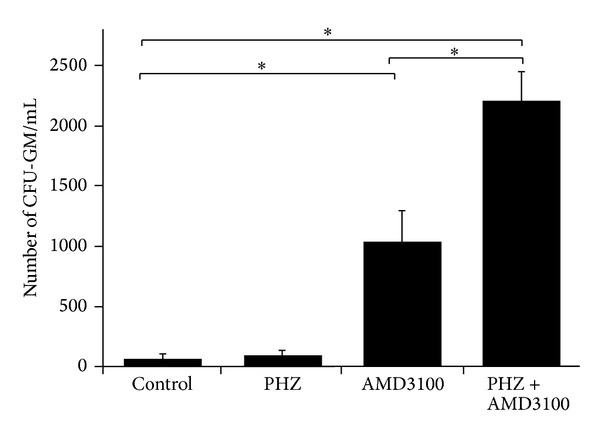
PHZ-induced mobilization of HSPCs is significantly potentiated after administration of AMD3100. The numbers of circulating CFU-GM able to grow colonies in methylcellulose cultures isolated from control, PHZ-, AMD3100-, and PHZ + AMD3100-injected C57Bl/6 mice are shown. The data are combined from two different experiments with 10 animals each. *P < 0.001.

Under steady-state conditions, the concentration of S1P in PB is already very high and, as we reported in the past [4, 10–12], is sufficient to chemoattract BM-residing HSPCs. During mobilization, however, the level of S1P may further increase due to release of S1P from erythrocytes and platelets following activation of the terminal part of the complement cascade. Even so, as shown in Figures 1 and 3, the increase in S1P level in PB induced only negligible egress of HSPCs from BM into PB compared with administration of AMD3100 (Figure 3). However, if AMD3100 was added following PHZ treatment, robust synergistic mobilization of HSPCs occurred (Figure 3).

Furthermore, we observed that, as previously described, the mobilization process is associated with activation of the CC, as confirmed by C5a ELISA, and an increase in the level of free hemoglobin (Hb) in PB, indicating generation of lytic C5b-C9 (MAC, Table 1). At the same time, we did not see significant changes in the overall level of plasma SDF-1, which was in the range of 0.5–1.5 ng/mL (data not shown), and therefore at a concentration that does not affect migration of HSPCs [4, 8].

## 4. Discussion

It is well known that hematopoietic stem/progenitor cells (HSPCs) circulate in peripheral blood (PB) and lymph during development, moving between major anatomical sites where hematopoiesis is initiated and/or temporarily active [13, 14]. Later in adult life, a small percentage of HSPCs is continuously released from BM niches into the PB, which may be envisioned as a highway by which HSPCs relocate between distant BM stem cell niches in order to keep the total pool of BM stem cells in balance. It has been demonstrated in mice that, under steady-state conditions, circulating HSPCs undergo a circadian rhythm in their circulation in PB, with the peak occurring early in the morning and the nadir at night [15].

The number of circulating HSPCs increases in response to (i) systemic or local inflammation, (ii) strenuous exercise, (iii) stress, and (iv) tissue/organ injury [13, 14]. The number of HSPCs in PB may increase up to 100-fold after administration of pharmacological agents that induce their forced egress into PB, a process known as “stem cell mobilization.” The most important mobilizing agents currently employed in the clinic are (i) cytokines (e.g., granulocyte colony stimulating factor; G-CSF), (ii) cytostatics (e.g., cyclophosphamide), (iii) CXCR4- or VLA-4-blocking molecules (AMD3100 or BIO4860, resp.), and (iv) certain chemokines (e.g., the growth-related oncogene protein-beta [Gro-*β*]) [13–19].

Pharmacological mobilization has been exploited in hematological transplantology as a means of obtaining HSPCs for hematopoietic reconstitution. HSPCs circulating in PB are currently a preferred source of stem cells for transplantation, because they are easily accessible and—what is important from a clinical point of view—in certain clinical situations, they are engrafted faster after transplantation than HSPCs harvested from the BM under steady-state conditions [13–19].

Several mechanisms have been proposed to orchestrate mobilization, but still more work is needed to better understand this process. Evidence is accumulating that the nature of mobilization varies with the mechanism that triggers or initiates it: systemic inflammation, tissue/organ injury, or pharmacological intervention. Moreover, every mobilizing drug may trigger mobilization by employing overlapping, yet different, mechanisms [13–19].

Overall, the mobilization process has been proposed to be directed by (i) a decrease in SDF-1–CXCR4 and VLA-4-VCAM-1 retention interactions in BM (e.g., due to release of proteolytic enzymes or molecular blockade after administration of small molecular antagonists) [13–19], (ii) release of neurotransmitters from the synapses of the nerves that innervate the BM microenvironment (e.g., involving the dopamine and *β*2-adrenergic receptors) [15], (iii) reversal of the transendothelial chemotactic gradient between the BM microenvironment and plasma [4], (iv) activation of the coagulation cascade (e.g., release of thrombin and uPAR) [9, 20], and finally, as recently proposed, (v) activation of the CC [13]. In particular, active products of the distal part of the CC, C5a and C5b-C9, are required for mobilization [21].

For many years it was assumed that the plasma level of SDF-1 was responsible for egress of HSPCs from BM into PB; however, as reported by several investigators, the SDF-1 level does not increase significantly during mobilization and thus does not explain the egress of HSPCs [4]. Recent research identifies sphingosine-1-phosphate (S1P) as a major chemoattractant for HSPCs already present in steady-state blood plasma [4, 10–12].

S1P is highly expressed in erythrocytes and can be released from these cells during hemolysis. In fact, hemolytic syndromes, such as SSA and PNH, are characterized by an elevated number of HSPCs circulating in PB [1–3]. However, the molecular mechanisms responsible for this effect are still not completely understood. Therefore, we focused on the potential role of S1P in this process.

In this paper, we demonstrate in an FHZ-induced hemolysis model that an increase in S1P plasma level alone is not sufficient to induce significant mobilization. This is not surprising, since, as we reported in the past, the plasma concentration of S1P under steady-state conditions is already high enough to chemoattract BM-residing HSPCs. To explain this observation, we postulated that retention of HSPCs is an active process that counteracts the effects of the S1P “chemotactic field” that is continuously present in PB plasma [4].

Our results described herein, in which we employed FHZ alone and FHZ + AMD3100 treatment, show that, in addition to increasing the S1P level in plasma, it is necessary to attenuate the retention mechanism of HSPCs in BM stem cell niches to ensure significant mobilization. Furthermore, increases in C5a level and free plasma Hb level as a result of generation of lytic C5b-C9 (MAC) provide further evidence for activation of the terminal part of the CC during the mobilization process [21, 22].

This result tends to support our observation of the mobilization of HSPCs during hemolytic episodes in patients suffering from PNH [3]. In these patients, mobilization occurs not only because S1P is released from the hemolysed erythrocytes, but also because PNH HSPC clones have defective retention in BM niches. Our data also support a recent previous report in which mice exposed both to S1P receptor agonist and AMD3100 showed increased mobilization of HSPCs compared with mice exposed to AMD3100 alone [11].

Based on our data, we conclude that hemolysis, even if it significantly elevates the S1P level in PB, requires attenuation of the CXCR4–SDF-1 axis-mediated retention of HSPCs in BM niches in order to affect mobilization of HSPCs. A full understanding of the mechanisms of stem cell mobilization in hemolytic syndromes will help to develop more efficient strategies for treating these disorders.

## Figures and Tables

**Table 1:** Activation of the complement cascade (CC) and increase in free hemoglobin (Hb) level in PB plasma after PHZ, AMD3100, and AMD3100 + PHZ administration.

	Control*	PHZ	AMD3100	PHZ + AMD3100
Activation of CC (increase in C5a level in PB plasma)	1.0	1.5 ± 0.2	1.4 ± 0.3	2.1 ± 0.2
Increase in free Hb level in PB plasma	1.0	1.4 ± 1.0	1.1 ± 0.4	1.3 ± 1.0

*Values in control mice were assumed to be 1.0.
